# From chronic inflammation to immune escape: mapping the tumor microenvironment evolution in renal cell carcinoma

**DOI:** 10.3389/fimmu.2025.1683844

**Published:** 2025-11-04

**Authors:** Hanjun Xu, Baojun Tu, Hui Li, Yong Shan

**Affiliations:** ^1^ Department of Urology, Taizhou Second People’s Hospital Affiliated to Yangzhou University, Taizhou, Jiangsu, China; ^2^ The First Clinical Medical College, Nanjing Medical University, Nanjing, Jiangsu, China; ^3^ Department of Nephrology, Taizhou Jiangyan Traditional Chinese Medicine Hospital, Jiangyan Affiliated Hospital of Nanjing University of Chinese Medicine, Taizhou, Jiangsu, China

**Keywords:** renal cell carcinoma, tumor immune microenvironment, chronic inflammation, IFN-γ signaling, immune exclusion, immunotherapy

## Abstract

Renal cell carcinoma (RCC) evolves within a chronic inflammatory renal niche, where angiogenesis, metabolism, and immune programs synergize to reshape the tumor immune microenvironment (TIME). Recurrent renal damage and tissue hypoxia sustain NF-κB/STAT3 and HIF-VEGF signaling, while a sustained IFN-γ response enhances antigen presentation while inducing inhibitory checkpoints, promoting a state of “inflammation but constrained.” Single-cell and spatial studies reveal early microenvironment heterogeneity and the chemokine-checkpoint paradox: regions enriched in CXCL9/10 coexist with endothelial inertia, cancer-associated fibroblasts (CAFs)-mediated stromal barriers, and metabolic stress, which collectively exclude functional CD8^+^T cells. In advanced clear cell RCC, immuno-inflammatory, immuno-excluded, and immuno-desert phenotypes often coexist and undergo transitions during treatment, leading to heterogeneity in response to immune checkpoint blockade (ICB). We propose a modular perspective-the NF-κB/STAT3, HIF-VEGF, IFN-γ circuits and auxiliary regulatory factors-to link stage-specific biology with treatment matching. Integrative biomarkers couple IFN-γ characteristics with angiogenesis/stromal modules and spatial indicators, offering superior predictive power compared to single tests. These insights support the adoption of a combined strategy: integrating vascular normalization or stromal/myeloid cell reprogramming on the basis of ICB, and encouraging the use of longitudinal “immune snapshots” to guide intervention and precision immunotherapy for renal cancer.

## 1 Introduction

Renal cell carcinoma (RCC) accounts for the vast majority of kidney malignancies and remains one of the leading causes of death from genitourinary system cancers globally. Its incidence rate is influenced by the increased exposure to metabolic and vascular risk factors, as well as the widespread use of cross-sectional imaging (1, 2). From a biological perspective, RCC exhibits significant inter- and intratumoral heterogeneity. Clear cell RCC (ccRCC) is the predominant subtype, characterized by the loss of the VHL gene and activation of the HIF pathway. Equally decisive is the ability of RCC to remodel and exploit the tumor immune microenvironment (TIME): tumor cells, stromal cells, and endothelial cell populations jointly regulate cytokine and chemokine programs, thereby refining lymphocyte migration, antigen presentation, and effector functions, gradually driving the transition from immune surveillance to immune evasion. The resulting immune ecology includes: immune infiltration regions accompanied by interferon response programs; immune exclusion zones where lymphocytes are sequestered at the edge due to fibrovascular and stromal barriers; and immune deserts with almost no leukocyte presence. These phenotypes are closely correlated with the heterogeneity of clinical behavior and differential responses to combined treatment with immune checkpoint blockade (ICB) and VEGF targeting (3, 4).

Under physiological conditions, the kidney maintains a relatively immune-tolerant and low-inflammatory environment to safeguard filtration and tubular transport, balancing constant antigen exposure with the need to avoid collateral tissue damage. This homeostasis is maintained by tissue-resident immune cells, such as macrophages, dendritic cells, and natural killer cells, which interact with tubular epithelial and endothelial cells to regulate antigen clearance and prevent excessive immune responses (5). However, this balance can be disrupted by persistent tissue injury, including chronic kidney disease, autoimmune nephritis, ischemia–reperfusion injury, metabolic stress, and smoldering microinflammation. These insults activate inflammatory pathways such as NF-κB, STAT3, and interferon-related signaling, leading to elevated chemokine expression, immune cell infiltration, and stromal remodeling. Together, these processes create an immune environment that supports tumor development (6). RCC originates and progresses within this altered immune context, gradually shifting from immune surveillance toward immune evasion.

During early tumorigenesis and the preneoplastic phase, interferon-γ (IFN-γ) signaling enhances antigen presentation components, such as MHC I/II, TAP1, and PSMB9. At the same time, it induces upregulation of inhibitory immune checkpoints, including PD-L1, VISTA, and B7-H3. These molecular changes are accompanied by the expansion of regulatory T cells and the emergence of dysfunctional CD8^+^T cells, collectively forming an “immune rewiring” state characterized by simultaneous immune activation and suppression (7). This immune remodeling shows considerable spatial heterogeneity, with immune-infiltrated regions coexisting with immune-excluded areas, where T cell access is limited by fibroblast-mediated matrix barriers, endothelial dysfunction, and metabolic constraints (8). As the tumor advances, the TIME evolves into distinct immune phenotypes such as T cell-inflamed, immune-excluded, and immune-desert. These phenotypes are not fixed but change dynamically under therapeutic pressure, which may explain the variable responses to ICB and VEGF-targeted therapies observed in RCC. Recognizing the spatiotemporal evolution of these immune states is crucial for identifying reliable biomarkers and guiding the development of adaptive immunotherapeutic strategies.

## 2 Chronic inflammation drives renal immune landscape remodeling

The healthy kidney maintains a low-inflammatory, immune-tolerant milieu to protect filtration and tubular transport. Persistent tissue injury, including chronic kidney disease, autoimmune nephritis, ischemia–reperfusion, metabolic stress, toxins, and smoldering micro-inflammation, can disrupt this balance and is associated with progressive rewiring of the renal immune niche (9, 10). These changes elevate chemokine production, recruit myeloid populations, and remodel stroma and endothelium, pre-positioning the renal TIME for immune editing (11, 12).

### 2.1 Core inflammatory modules and effector programs

Three interacting modules organize this transition and reinforce one another. First, innate sensors and alarmins sustain NF-κB/STAT3 signaling; complement and inflammasome pathways further amplify myeloid-biased inflammation and promote tolerogenic dendritic-cell states with low CCR7/CD86, limiting effective T-cell priming and egress (13, 14). Second, capillary rarefaction and fibrosis drive tubulointerstitial hypoxia, stabilizing HIF-1/2 and amplifying VEGF-dependent angiogenic programs that reshape vascular structure and trafficking cues (15–17). Third, IFN-γ signaling is a double-edged module: it enhances antigen-presentation machinery while concurrently inducing inhibitory checkpoints and fostering dysfunctional/exhausted T-cell states under chronic exposure (18, 19). In parallel, cancer-associated fibroblasts (CAFs) and activated endothelium remodel matrix and vasculature to limit lymphocyte access, consolidating an inflamed-yet-restrained program (20).

### 2.2 Cellular and stromal remodeling in chronically inflamed kidneys

Chronically inflamed kidneys undergo coordinated remodeling of immune and stromal compartments. Neutrophils accumulate and release elastase and neutrophil extracellular traps (NETs), injuring epithelium and potentially unmasking neoantigens (21). Monocyte-derived macrophages polarize toward M2-like states that secrete IL-10, TGF-β, and prostaglandins. Dendritic cells remain functionally immature, limiting effective T-cell priming and egress, while natural killer cells exhibit reduced cytotoxicity (22). CD4^+^T cells skew toward Th17 and Treg subsets, and CD8^+^T cells display early dysfunction/exhaustion markers despite increased antigen exposure (23). In parallel, fibroblasts acquire CAF-like phenotypes, produce CXCL12, and reorganize collagen, creating physical and chemotactic barriers; the vasculature becomes abnormal and anergic, with reduced expression of adhesion molecules such as ICAM-1/VCAM-1 that impair leukocyte trafficking. Animal models, including ischemia–reperfusion injury, adenine or folate nephropathy, and chemical carcinogen exposure, recapitulate these features, with progressive vascular dysfunction, checkpoint induction, and myeloid dominance preceding overt tumorigenesis (24, 25). Human bulk and single-cell datasets from diseased kidneys and early RCC similarly capture these transitions, linking chronic inflammation to tumor development and suggesting a tractable pre-malignant window in which anti-cytokine, vascular-normalizing, and stroma- or myeloid-targeted interventions may help restore immune surveillance and delay or prevent RCC initiation (26). Therapeutic options aligned to these modules are outlined in **Table 1**.

**Table 1:** Modular view of chronic-inflammation–driven remodeling of the renal tumor immune microenvironment (TIME) and therapeutic implications in RCC.

Module	Key pathways/effectors	TIME consequences	Example combination strategies
NF-κB/STAT3 axis	Persistent cytokines/alarmins; DAMPs; microbial products TLRs; cGAS–STING; IL-1R/IL-6R→JAK; NF-κB/STAT3 IL-1β, IL-6, TNF-α; CCL2; CXCL1/8/12; SASP factors	Chronic myeloid recruitment; tolerogenic DCs; sustained inflammatory and pro-survival signaling (13, 14)	IL-1/IL-6 or JAK/STAT blockade as ICB partners
Complement & inflammasome	Tissue injury/ischemia; crystals; infection C3/C5 axis; NLRP3–caspase-1; IL-1R/IL-18R C3a, C5a; IL-1β, IL-18	Myeloid activation; endothelial dysfunction; DC impairment (13, 14)	C5aR/C3 inhibitors; IL-1 blockade as rational partners to ICB
Hypoxia–HIF–VEGF	Capillary rarefaction; fibrosis; VHL loss; tubulointerstitial hypoxia HIF-1α/2α stabilization; VEGF/VEGFR; ANGPT–TIE VEGF-A; ANGPT2; CA9	Leaky/abnormal vessels; endothelial anergy; impaired T-cell trafficking (15, 16)	Anti-VEGF/VEGFR or anti-ANGPT2; vascular normalization combined with ICB
IFN-γ program (double-edged)	Chronic antigen exposure; tonic cytokine loops IFN-γ-induced JAK/STAT1/IRF1 axis upregulating both antigen presentation and checkpoint molecules MHC I/II, TAP1, PSMB9; PD-L1, VISTA, B7H3	Enhanced antigen presentation but checkpoint induction; progressive T-cell dysfunction under chronic stimulation (18)	ICB ± co-stimulation/IL-2 variants; composite biomarkers coupling IFN-γ with angiogenic/stromal modules
TGF-β/CAF & ECM remodeling	Chronic injury; stiff ECM; hypoxia TGF-β/SMAD; PDGFR; FAK; LOX Collagen I/III, fibronectin; CXCL12; periostin; α-SMA	ECM deposition/crosslinking; CAF barriers; immune exclusion (20)	TGF-β/CAF targeting; FAK or CXCL12–CXCR4 axis inhibition + ICB

## 3 Immune rewiring in preneoplastic/early RCC

### 3.1 IFN-γ–driven epithelial responses and early checkpoint induction

In the preneoplastic and early stages of RCC, persistent IFN-γ signaling—mainly derived from tissue-resident and infiltrating T lymphocytes, amplifies antigen processing and presentation on the one hand, and installs “molecular brakes” for effector activities on the other hand. IFN-γ activates the STAT1/IRF1 pathway, which upregulates classical MHC-I molecules and key regulators such as NLRC5 and B2M. It also induces MHC-II expression through CIITA, enhancing tumor antigen presentation to CD4^+^ T cells (27). Pattern-recognition pathways, including RIG-I/MDA5 and the cGAS-STING axis, can via type I/II interferon signaling, reinforce antigen-processing and presentation programs.

However, this enhanced immunogenic state is accompanied by early and coordinated expression of inhibitory immune checkpoints including PD-L1, VISTA, B7-H3, and occasionally B7-H4, across epithelial, endothelial, and myeloid cell populations. These molecules together form a suppressive network that limits effective T cell co-stimulation (28). In parallel, the tumor epithelium undergoes metabolic reprogramming that dampens immune responses. For example, IDO1 and TDO2 facilitate kynurenine production, activating the aryl hydrocarbon receptor (AHR); extracellular adenosine accumulates via CD39/CD73; and elevated glycolytic activity leads to lactic acid buildup—all of which impair cytotoxic T cell function and antigen processing (29). Although these early changes may serve as a protective adaptation, they gradually favor the survival of epithelial cell clones resistant to IFN signaling, promoting immune editing. With tumor progression, temporary regulatory adaptations, such as PD-L1 promoter acetylation or inducible MHC-II expression, are often replaced by irreversible genetic alterations, including loss of HLA heterozygosity, disruption of B2M, and mutations in JAK-STAT signaling components, leading to a fixed immune escape phenotype (30). This “inflamed but restrained” immune state represents a common bottleneck in the early evolution of RCC and is increasingly recognized as a window for therapeutic intervention.

### 3.2 Spatial and single-cell insights: heterogeneity emerges early

Recent advances in spatial transcriptomics and single-cell RNA sequencing have revealed that, even at early stages of ccRCC, the TIME is highly heterogeneous at the tissue level. Immune-active “inflammatory islands” can be found next to immunosuppressive regions within the same tumor. The former are characterized by IRF1/STAT1 signaling, secretion of CXCL9/10, and inducible expression of HLA class II, while the latter show features such as TGF-β signaling, EMT activation, checkpoint ligand expression, and impaired antigen processing (31). This spatial pattern is partly related to epithelial lineage, where proximal tubule-like cells retain stronger immune visibility, whereas distal or collecting duct-like phenotypes tend toward immune evasion (32). The immune cells reflect this organization: Tregs accumulate near active niches, while CD8^+^ T cells divide into precursor exhausted and terminally exhausted subsets, the latter enriched in metabolically stressed areas with high lactate and adenosine (33). The number of conventional dendritic cells was maintained but the function was immature, which limited the effective T-cell priming and egress. Although plasmacytoid and monocyte-derived DCs express interferon-stimulating genes in varying degrees, they do not perform well in cross presentation. Meanwhile, macrophages polarize toward an M2-like state, producing IL-10 and TGF-β, which promote immune suppression and activate CAFs. These CAFs further diversify into inflammatory and myofibroblastic types, with the latter forming aligned collagen that directs but restricts CD8^+^T cell entry. Tumor-associated endothelial cells also vary, with stalk-like cells lacking HEV features and expressing low ICAM-1/VCAM-1, disrupting immune cell trafficking (34). TLS may form near inflammatory zones, containing B and T cells with germinal center markers. Their function depends on context—supportive when antigen presentation is active and matrix barriers are limited, but potentially tolerogenic when CAFs and suppressive myeloid cells dominate. These high-resolution data explain why the overall detection can show “inflammatory type”, while the lesions are “unresponsive”: the inflammation and immune escape microregions are staggered on the sub millimeter scale, and their relative proportion rather than simple existence determines the function.

### 3.3 Chemokine–checkpoint paradox and early risk stratification

A recurring feature of early RCC evolution is the chemokine checkpoint paradox: Although the level of chemokine that attracts T cells is very high, the degree of truly effective CD8^+^ entering the epithelial “nest” is limited. This leads to a pattern of spatial immune exclusion despite a seemingly inflamed transcriptional profile. Several mechanisms contribute to this paradox: (i) Matrix gating: CXCL12 produced by CAF forms gradients that trap CXCR4^+^ T cells in perivascular regions, while ECM remodeling through collagen I/III deposition increases tissue stiffness and pressure, limiting T-cell movement. (II) Endothelium unresponsive: high VEGF, HIF-dependent vascular down-regulation of adhesion molecules, abnormal pericyte coating, and the expression of FasL or PD-L1, collectively scavenge or inactivate effector T cells during transendothelial migration. (III) Metabolism/ion barrier: lactate accumulation, hypoxia and adenosine signaling reduce T cell motility, cytokine production and survival; Potassium leakage from dying cells disrupts T cell translation and biosynthesis. (IV) Premature checkpoint tone: epithelial and myeloid cells express PD-L1/TIM-3 ligand, and myeloid/endothelial cells express VISTA, which inactivates the effector function just at the “entrance” indicated by chemotactic signals. As a result, the TIME appears immunologically active at the transcript level but functionally excludes effector T cells (35). For clinical application, single biomarkers like PD-L1 staining are often insufficient. Instead, integrated models that combine IFN-γ response signatures, co-expression of inhibitory receptors, myeloid/CAF-derived suppressive signals, and spatial parameters offer a more accurate basis for risk assessment (8). These multidimensional approaches can help identify lesions with high progression risk and support early intervention strategies—such as vascular or ECM normalization to facilitate immune entry, tailored checkpoint blockade to prevent early tolerance, and myeloid reprogramming to restore responsiveness—before RCC becomes clinically evident.

## 4 Established RCC: from immune dysfunction to clinical translation

**Figure 1:**
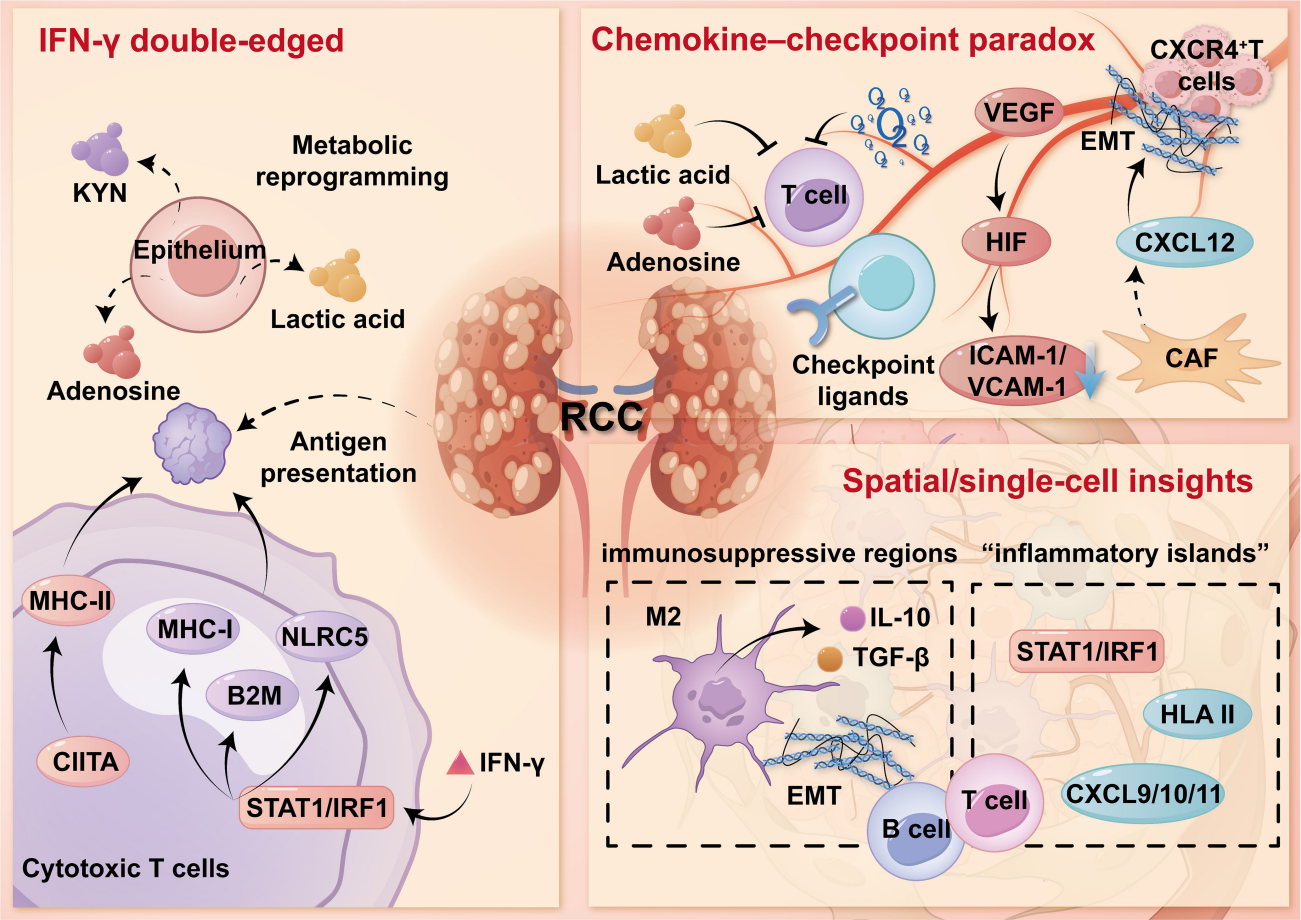
Chronic inflammation primes RCC for an “inflamed-yet-restrained” TIME.

In established ccRCC, the TIME typically presents as one of three immune phenotypes, including immune-infiltrated, immune-excluded, or immune-desert, each defined by distinct immune cell profiles, spatial patterns, and functional states. Immune-infiltrated tumors contain abundant intratumoral CD8^+^T cells, activated DCs, and elevated interferon signaling, reflecting an engaged, though often exhausted, immune response. In immune-excluded tumors, immune cells are present but confined to the tumor periphery or fibrotic stromal zones. Infiltration is limited by endothelial dysfunction, ECM barriers, and suppressive cytokines like TGF-β and VEGF, commonly shaped by CAF activity and hypoxia (36). Desert tumors, by contrast, show minimal immune infiltration, low MHC expression, and weak chemokine activity. These are often associated with low tumor mutation burden and are generally resistant to ICB therapies. Spatial transcriptomics and multiplex imaging have revealed that these phenotypes can coexist within the same tumor, with sharp transitions between inflamed and excluded areas at sub-millimeter scales (37). This spatial diversity helps explain the frequent presence of mixed immune regions in a single lesion. Moreover, TIME phenotypes are not fixed. They shift over time in response to tumor-intrinsic factors like hypoxia or loss of BAP1 and CDKN2A/B, as well as external pressures from therapy. Longitudinal studies have shown, for example, that inflamed tumors may transition to excluded phenotypes after ICB due to immune exhaustion or stromal remodeling, while anti-VEGF therapy may restore vascular function and allow T cell entry (38). These insights support the view that TIME phenotypes are dynamic and modifiable, reinforcing the need for flexible, time-adapted immunotherapy strategies in RCC treatment. Collectively, **Figure 1** integrates epithelial IFN-γ responses, vascular/stromal constraints, and early spatial heterogeneity, providing a mechanistic basis for response prediction and for combination strategies pairing ICB with vascular normalization and stromal/myeloid reprogramming.

### 4.1 T-cell dysfunction, myeloid/CAF suppression, and hypoxia coupling

In the TIME of established ccRCC, CD8^+^T cells often remain dysfunctional despite their presence in adequate numbers, reflecting progression along an exhaustion spectrum. These cells range from progenitor-like to terminally exhausted states, characterized by diminished cytotoxic potential, reduced granzyme and perforin production, impaired mitochondrial biogenesis, and metabolic stress driven by lactate accumulation and lipid overload (39). Persistent antigen stimulation activates NFAT-dependent TOX expression, reinforcing epigenetic programs that lock in the exhausted phenotype. At the same time, immunosuppressive myeloid populations—including M2-polarized macrophages and both granulocytic and monocytic MDSCs—produce suppressive mediators such as NO, ROS, arginase, IL-10, and TGF-β. These factors inhibit T cell priming and cytotoxicity while impairing DC maturation and cross-priming (40). CAFs contribute further by depositing aligned collagen fibers and releasing CXCL12, which traps CXCR4^+^T cells in perivascular zones, forming both structural and chemotactic barriers to infiltration. In parallel, VEGF and hypoxia induce endothelial anergy by reducing ICAM-1/VCAM-1 expression and altering pericyte coverage. Some endothelial cells additionally express FasL and PD-L1, directly eliminating incoming T cells. Hypoxia also stabilizes HIF-1α/2α, promoting VEGF-driven but inefficient angiogenesis that sustains acidosis and nutrient deprivation, ultimately limiting T cell survival and motility. Notably, recent studies show that chronic antigen exposure under hypoxic conditions induces rapid terminal exhaustion of CD8^+^T cells via mitochondrial ROS accumulation and suppression of PGC-1α signaling, linking metabolic stress to immunotherapy resistance. Taken together, the interplay between intrinsic T cell exhaustion, suppressive myeloid and stromal elements, and hypoxia-induced metabolic dysfunction creates a TIME that strongly predicts poor response to immune checkpoint blockade, unless these barriers—particularly stromal and vascular components—are actively reprogrammed (41).

### 4.2 Clinical translation and therapeutic matching

Around the dominant TIME phenotype matching therapy, the response probability and persistence can be improved at the same time. Immune-infiltrated tumors, marked by pre-existing TILs, intact antigen presentation, and interferon signaling, respond well to PD-1/PD-L1 inhibitors, either alone or combined with CTLA-4 blockade. In contrast, immune-excluded tumors—often shaped by abnormal angiogenesis and dysfunctional endothelium—may benefit more from combining ICB with VEGF/VEGFR inhibitors. These agents can restore vascular integrity, upregulate ICAM-1/VCAM-1, reduce myeloid infiltration, and enhance T cell entry. Immune-desert tumors, or those dominated by suppressive myeloid cells and CAFs, may require additional interventions targeting pathways, alongside strategies to reduce ECM stiffness or metabolic constraints, in order to create a more immune-permissive environment prior to or together with checkpoint inhibition (42). Biomarker strategies that combine indices of T cell inflammation with angiogenic and stromal signatures offer better predictive power than single markers like PD-L1 IHC. In cases with unclear immune profiles, genomic alterations in chromatin remodeling or DNA repair genes may further refine patient selection. Clinical management should also be adaptive, with immune phenotype shifts tracked by serial biopsy or validated surrogates like imaging-based assessments of perfusion or immune activity. For example, adding anti-angiogenic or myeloid-targeting agents may help shift immune-excluded tumors toward inflamed states, while inflamed tumors may remain responsive to ICB alone. Current RCC trials increasingly stratify patients by TIME phenotype and dominant resistance mechanism and define success not only by traditional endpoints but also by biological transitions such as reversal of immune exclusion, aiming to reprogram the tumor microenvironment and restore cytotoxic immunity.

## 5 Discussion

Despite major advances in immune profiling technologies, turning static immune “snapshots” into clinically useful biomarkers in RCC remains challenging. Technical factors—such as biopsy location, ischemia time, and fixation protocols—can introduce variability, while differences between profiling platforms and downstream analysis pipelines often result in inconsistent interpretations of immune cell states (43). RCC is also highly spatially heterogeneous: a single core can miss key features of the TIME, from inflamed pockets to immune-excluded zones or myeloid/CAF-dominated areas. Time compounds the problem. Therapy, intercurrent infection, or ischemia-reperfusion can quickly remodel the TIME, so a baseline sample rarely captures true dynamics. Cross-platform integration remains immature: scRNA-seq, spatial transcriptomics, multiplex IHC/IMC, and digital pathology each show a different slice of biology, and we lack robust reference atlases and external validation to fuse them into stable, generalizable classifiers. Clinical qualification is another hurdle: assays must show analytical validity, clinical validity, and real utility, within the constraints of trial timelines, budgets, and regulators. Meaningful progress will require harmonized SOPs, multi-region and longitudinal sampling, built-in QC, and prospective replication in diverse cohorts before immune snapshots can routinely guide RCC care (26).

Looking ahead, progress in RCC immunotherapy will depend on adopting a longitudinal, multi-modal approach that integrates spatial, single-cell, and histopathologic data to monitor transitions between inflamed, excluded, and desert TIME states. Trial stratification should consider both immune phenotype and dominant resistance pathways—whether checkpoint-driven, angiogenic, or myeloid/CAF-mediated—to guide rational combinations. Adaptive trial designs with dynamic treatment adjustment based on TIME remodeling could improve therapeutic sequencing. Endpoints should move beyond traditional metrics like ORR and PFS to capture meaningful biological transitions, such as exclusion-to-inflamed conversion or restoration of myeloid–lymphoid balance, supported by real-time biomarkers. Developing standardized immune atlases, validated surrogate markers, and accessible high-fidelity assays will be essential to enable clinical translation. Ultimately, refining immune classification tools and matching therapies to the underlying resistance mechanisms will allow immune snapshots to serve not just as static readouts but as real-time guides for interception, sequencing, and combination treatment in RCC.

## References

[1] Bukavina L Bensalah K Bray F Carlo M Challacombe B Karam JA. Epidemiology of renal cell carcinoma: 2022 update. Eur Urol. (2022) 82:529–42. doi: 10.1016/j.eururo.2022.08.019, PMID: 36100483

[2] Makino T Kadomoto S Izumi K Mizokami A. Epidemiology and prevention of renal cell carcinoma. Cancers (Basel). (2022) 14(16):4059. doi: 10.3390/cancers14164059, PMID: 36011051 PMC9406474

[3] Zheng W Zhang S Guo H Chen X Huang Z Jiang S. Multi-omics analysis of tumor angiogenesis characteristics and potential epigenetic regulation mechanisms in renal clear cell carcinoma. Cell communication signaling: CCS. (2021) 19:39. doi: 10.1186/s12964-021-00728-9, PMID: 33761933 PMC7992844

[4] Chappell JC Payne LB Rathmell WK. Hypoxia, angiogenesis, and metabolism in the hereditary kidney cancers. J Clin Invest. (2019) 129:442–51. doi: 10.1172/jci120855, PMID: 30614813 PMC6355237

[5] Qu Z Chu J Jin S Yang C Zang J Zhang J. Tissue-resident macrophages and renal diseases: landscapes and treatment directions. Front Immunol. (2025) 16:1548053. doi: 10.3389/fimmu.2025.1548053, PMID: 40230850 PMC11994677

[6] Kruk L Mamtimin M Braun A Anders HJ Andrassy J Gudermann T. Inflammatory networks in renal cell carcinoma. Cancers (Basel). (2023) 15(8):2212. doi: 10.3390/cancers15082212, PMID: 37190141 PMC10136567

[7] Qin S Jiang J Lu Y Nice EC Huang C Zhang J. Emerging role of tumor cell plasticity in modifying therapeutic response. Signal transduction targeted Ther. (2020) 5:228. doi: 10.1038/s41392-020-00313-5, PMID: 33028808 PMC7541492

[8] Bruni S Mercogliano MF Mauro FL Cordo Russo RI Schillaci R. Cancer immune exclusion: breaking the barricade for a successful immunotherapy. Front Oncol. (2023) 13:1135456. doi: 10.3389/fonc.2023.1135456, PMID: 37284199 PMC10239871

[9] Arabi T Shafqat A Sabbah BN Ashraf N Shah H Abdulkader H. Obesity-related kidney disease: beyond hypertension and insulin-resistance. Front Endocrinol. (2022) 13:1095211. doi: 10.3389/fendo.2022.1095211, PMID: 36726470 PMC9884830

[10] Oda Y Nishi H Nangaku M. Role of inflammation in progression of chronic kidney disease in type 2 diabetes mellitus: clinical implications. Semin Nephrol. (2023) 43:151431. doi: 10.1016/j.semnephrol.2023.151431, PMID: 37865982

[11] Kim J Ha S Son M Kim D Kim MJ Kim B. Tlr7 activation by Mir-21 promotes renal fibrosis by activating the pro-inflammatory signaling pathway in tubule epithelial cells. Cell communication signaling: CCS. (2023) 21:215. doi: 10.1186/s12964-023-01234-w, PMID: 37596656 PMC10439664

[12] Chung KW Dhillon P Huang S Sheng X Shrestha R Qiu C. Mitochondrial damage and activation of the sting pathway lead to renal inflammation and fibrosis. Cell Metab. (2019) 30:784–99.e5. doi: 10.1016/j.cmet.2019.08.003, PMID: 31474566 PMC7054893

[13] Zhao H Wu L Yan G Chen Y Zhou M Wu Y. Inflammation and tumor progression: signaling pathways and targeted intervention. Signal transduction targeted Ther. (2021) 6:263. doi: 10.1038/s41392-021-00658-5, PMID: 34248142 PMC8273155

[14] Kourtis N Wang Q Wang B Oswald E Adler C Cherravuru S. A single-cell map of dynamic chromatin landscapes of immune cells in renal cell carcinoma. Nat Cancer. (2022) 3:885–98. doi: 10.1038/s43018-022-00391-0, PMID: 35668194 PMC9325682

[15] Liu ZJ Semenza GL Zhang HF. Hypoxia-inducible factor 1 and breast cancer metastasis. J Zhejiang Univ Sci B. (2015) 16:32–43. doi: 10.1631/jzus.B1400221, PMID: 25559953 PMC4288942

[16] Hoefflin R Harlander S Schäfer S Metzger P Kuo F Schönenberger D. Hif-1α and Hif-2α Differently regulate tumor development and inflammation of clear cell renal cell carcinoma in mice. Nat Commun. (2020) 11:4111. doi: 10.1038/s41467-020-17873-3, PMID: 32807776 PMC7431415

[17] Motz GT Santoro SP Wang LP Garrabrant T Lastra RR Hagemann IS. Tumor endothelium fasl establishes a selective immune barrier promoting tolerance in tumors. Nat Med. (2014) 20:607–15. doi: 10.1038/nm.3541, PMID: 24793239 PMC4060245

[18] Jorgovanovic D Song M Wang L Zhang Y. Roles of ifn-Γ in tumor progression and regression: A review. biomark Res. (2020) 8:49. doi: 10.1186/s40364-020-00228-x, PMID: 33005420 PMC7526126

[19] Abiko K Matsumura N Hamanishi J Horikawa N Murakami R Yamaguchi K. Ifn-Γ from lymphocytes induces Pd-L1 expression and promotes progression of ovarian cancer. Br J Cancer. (2015) 112:1501–9. doi: 10.1038/bjc.2015.101, PMID: 25867264 PMC4453666

[20] Salminen A Kaarniranta K Kauppinen A. Tissue fibroblasts are versatile immune regulators: an evaluation of their impact on the aging process. Ageing Res Rev. (2024) 97:102296. doi: 10.1016/j.arr.2024.102296, PMID: 38588867

[21] Xie R Shang B Shi H Bi X Song Y Qu W. Neutrophil extracellular traps in relationship to efficacy of systemic therapy for metastatic renal cell carcinoma. Cancer Med. (2023) 12:21807–19. doi: 10.1002/cam4.6748, PMID: 38018346 PMC10757093

[22] Li J Yang Y Wang Y Li Q He F. Metabolic signatures of immune cells in chronic kidney disease. Expert Rev Mol Med. (2022) 24:e40. doi: 10.1017/erm.2022.35, PMID: 36268748 PMC9884772

[23] Li L Yang C Zhao Z Xu B Zheng M Zhang C. Skewed T-helper (Th)1/2- and Th17/T regulatory−Cell balances in patients with renal cell carcinoma. Mol Med Rep. (2015) 11:947–53. doi: 10.3892/mmr.2014.2778, PMID: 25352158 PMC4262517

[24] Guzzi F Cirillo L Roperto RM Romagnani P Lazzeri E. Molecular mechanisms of the acute kidney injury to chronic kidney disease transition: an updated view. Int J Mol Sci. (2019) 20(19):4941. doi: 10.3390/ijms20194941, PMID: 31590461 PMC6801733

[25] Jaworska K Ratajczak J Huang L Whalen K Yang M Stevens BK. Both Pd-1 ligands protect the kidney from ischemia reperfusion injury. J Immunol. (2015) 194:325–33. doi: 10.4049/jimmunol.1400497, PMID: 25404361 PMC4272874

[26] Borcherding N Vishwakarma A Voigt AP Bellizzi A Kaplan J Nepple K. Mapping the immune environment in clear cell renal carcinoma by single-cell genomics. Commun Biol. (2021) 4:122. doi: 10.1038/s42003-020-01625-6, PMID: 33504936 PMC7840906

[27] Shukla A Cloutier M Appiya Santharam M Ramanathan S Ilangumaran S. The Mhc class-I transactivator Nlrc5: implications to cancer immunology and potential applications to cancer immunotherapy. Int J Mol Sci. (2021) 22(4):1964. doi: 10.3390/ijms22041964, PMID: 33671123 PMC7922096

[28] Mielcarska S Kot A Dawidowicz M Kula A Sobków P Kłaczka D. B7-H3 in cancer immunotherapy-prospects and challenges: A review of the literature. Cells. (2025) 14(15):1209. doi: 10.3390/cells14151209, PMID: 40801642 PMC12346708

[29] Labadie BW Bao R Luke JJ. Reimagining Ido pathway inhibition in cancer immunotherapy via downstream focus on the tryptophan-kynurenine-aryl hydrocarbon axis. Clin Cancer Res. (2019) 25:1462–71. doi: 10.1158/1078-0432.Ccr-18-2882, PMID: 30377198 PMC6397695

[30] Wang H Fu C Du J Wang H He R Yin X. Enhanced histone H3 acetylation of the Pd-L1 promoter via the Cop1/C-Jun/Hdac3 axis is required for Pd-L1 expression in drug-resistant cancer cells. J Exp Clin Cancer Res. (2020) 39:29. doi: 10.1186/s13046-020-1536-x, PMID: 32024543 PMC7003365

[31] Song X Zhu Y Geng W Jiao J Liu H Chen R. Spatial and single-cell transcriptomics reveal cellular heterogeneity and a novel cancer-promoting Treg cell subset in human clear-cell renal cell carcinoma. J immunotherapy Cancer. (2025) 13(1):e010183. doi: 10.1136/jitc-2024-010183, PMID: 39755578 PMC11748785

[32] Yu Z Lv Y Su C Lu W Zhang R Li J. Integrative single-cell analysis reveals transcriptional and epigenetic regulatory features of clear cell renal cell carcinoma. Cancer Res. (2023) 83:700–19. doi: 10.1158/0008-5472.Can-22-2224, PMID: 36607615 PMC9978887

[33] Yang G Cheng J Xu J Shen C Lu X He C. Metabolic heterogeneity in clear cell renal cell carcinoma revealed by single-cell Rna sequencing and spatial transcriptomics. J Transl Med. (2024) 22:210. doi: 10.1186/s12967-024-04848-x, PMID: 38414015 PMC10900752

[34] Krishna C DiNatale RG Kuo F Srivastava RM Vuong L Chowell D. Single-cell sequencing links multiregional immune landscapes and tissue-resident T cells in ccrcc to tumor topology and therapy efficacy. Cancer Cell. (2021) 39:662–77.e6. doi: 10.1016/j.ccell.2021.03.007, PMID: 33861994 PMC8268947

[35] Jerby-Arnon L Shah P Cuoco MS Rodman C Su MJ Melms JC. A cancer cell program promotes T cell exclusion and resistance to checkpoint blockade. Cell. (2018) 175:984–97.e24. doi: 10.1016/j.cell.2018.09.006, PMID: 30388455 PMC6410377

[36] Zheng S Wang W Shen L Yao Y Xia W Ni C. Tumor battlefield within inflamed, excluded or desert immune phenotypes: the mechanisms and strategies. Exp Hematol Oncol. (2024) 13:80. doi: 10.1186/s40164-024-00543-1, PMID: 39107856 PMC11301948

[37] Sobottka B Vetter V Banaei-Esfahani A Nowak M Lorch A Sirek A. Immune phenotype-genotype associations in primary clear cell renal cell carcinoma and matched metastatic tissue. Modern Pathol. (2024) 37:100558. doi: 10.1016/j.modpat.2024.100558, PMID: 38969270

[38] Tsuzuki T Ohe C Osawa T Yasuda Y Tanaka T Anai S. Prognostic value of immune phenotype and Pd-L1 status in recurrent or metastatic renal cell carcinoma: an exploratory analysis of the archery study. Pathology. (2023) 55:31–9. doi: 10.1016/j.pathol.2022.07.013, PMID: 36241555

[39] Pichler R Siska PJ Tymoszuk P Martowicz A Untergasser G Mayr R. A chemokine network of T cell exhaustion and metabolic reprogramming in renal cell carcinoma. Front Immunol. (2023) 14:1095195. doi: 10.3389/fimmu.2023.1095195, PMID: 37006314 PMC10060976

[40] Annels NE Denyer M Nicol D Hazell S Silvanto A Crockett M. The dysfunctional immune response in renal cell carcinoma correlates with changes in the metabolic landscape of ccrcc during disease progression. Cancer Immunol Immunother. (2023) 72:4221–34. doi: 10.1007/s00262-023-03558-5, PMID: 37940720 PMC10700462

[41] Scharping NE Rivadeneira DB Menk AV Vignali PDA Ford BR Rittenhouse NL. Mitochondrial stress induced by continuous stimulation under hypoxia rapidly drives T cell exhaustion. Nat Immunol. (2021) 22:205–15. doi: 10.1038/s41590-020-00834-9, PMID: 33398183 PMC7971090

[42] Wu F Yang J Liu J Wang Y Mu J Zeng Q. Signaling pathways in cancer-associated fibroblasts and targeted therapy for cancer. Signal transduction targeted Ther. (2021) 6:218. doi: 10.1038/s41392-021-00641-0, PMID: 34108441 PMC8190181

[43] Lyskjær I Iisager L Axelsen CT Nielsen TK Dyrskjøt L Fristrup N. Management of renal cell carcinoma: promising biomarkers and the challenges to reach the clinic. Clin Cancer Res. (2024) 30:663–72. doi: 10.1158/1078-0432.Ccr-23-1892, PMID: 37874628 PMC10870122

